# Maternal weight latent trajectories and associations with adverse pregnancy outcomes using a smoothing mixture model

**DOI:** 10.1038/s41598-023-36312-z

**Published:** 2023-06-02

**Authors:** Shirin Faraji Azad, Akbar Biglarian, Maryam Rostami, Razieh Bidhendi-Yarandi

**Affiliations:** 1 Department of Biostatistics and Epidemiology, University of Social Welfare and Rehabilitation Sciences, Tehran, Iran; 2 Social Determinants of Health Research Center, University of Social Welfare and Rehabilitation Sciences, Tehran, Iran; 3 Department of Community Medicine, Faculty of Medicine, Ahvaz Jundishapur University of Medical Sciences, Ahvaz, Iran

**Keywords:** Epidemiology, Outcomes research, Paediatric research

## Abstract

Class membership is a critical issue in health data sciences. Different types of statistical models have been widely applied to identify participants within a population with heterogeneous longitudinal trajectories. This study aims to identify latent longitudinal trajectories of maternal weight associated with adverse pregnancy outcomes using smoothing mixture model (SMM). Data were collected from the Khuzestan Vitamin D Deficiency Screening Program in Pregnancy. We applied the data of 877 pregnant women living in Shooshtar city, whose weights during the nine months of pregnancy were available. In the first step, maternal weight was classified and participants were assigned to only one group for which the estimated trajectory is the most similar to the observed one using SMM; then, we examined the associations of identified trajectories with risk of adverse pregnancy endpoints by applying logistic regression. Three latent trajectories for maternal weight during pregnancy were identified and named as low, medium and high weight trajectories. Crude estimated odds ratio (OR) for icterus, preterm delivery, NICU admission and composite neonatal events shows significantly higher risks in trajectory 1 (low weight) compared to trajectory 2 (medium weight) by 69% (OR = 1.69, 95%CI 1.20, 2.39), 82% (OR = 1.82, 95%CI 1.14, 2.87), 77% (OR = 1.77, 95%CI 1.17, 2.43), and 85% (OR = 1.85, 95%CI 1.38, 2.76), respectively. Latent class trajectories of maternal weights can be accurately estimated using SMM. It is a powerful means for researchers to appropriately assign individuals to their class. The U-shaped curve of association between maternal weight gain and risk of maternal complications reveals that the optimum place for pregnant women could be in the middle of the growth curve to minimize the risks. Low maternal weight trajectory compared to high had even a significantly higher hazard for some neonatal adverse events. Therefore, appropriate weight gain is critical for pregnant women.

*Trial registration* International Standard Randomized Controlled Trial Number (ISRCTN): 2014102519660N1; http://www.irct.ir/searchresult.php?keyword=&id=19660&number=1&prt=7805&total=10&m=1 (Archived by WebCite at http://www.webcitation.org/6p3lkqFdV).

## Introduction

Identifying latent longitudinal trajectories has become very popular in health sciences. Longitudinal trajectories are mostly provided by repeated observations and estimated by mixed-effects models^1^. They capture the individual variations over time and make a group membership for each participant. Later the potential effect of heterogeneous trajectories on the outcomes could be measured. The issue of patient-reported outcome (PRO) responses to treatment over time in heterogeneous clinical populations^2^, adolescent smoking trajectories^3^, trajectories of multi-morbidity in primary care^4^, and dose–response and pain trajectories^5,6^ are examples showing that there exist unobserved subgroups of patients within the population that indicate variability in their treatment responses and have heterogeneous health phenotypes.

Different types of statistical approaches have been used to assign participants within a population with heterogeneous longitudinal trajectories called generalized linear mixed models (GLMMs).

Generally, GLMMs apply a unified likelihood approach in order to parametrically model covariate effects, considering overdispersion and correlation by adding random effects to the linear predictor. However, a full likelihood analysis in GLMMs is burdensome due to intractable numerical integration^7^. In addition, the parametric mean assumption may not always be acceptable due to the unknown functional forms of the covariates and complicated association of the outcome variable and the covariates. Hence, a nonparametric regression model for correlated data is desirable as it allows more flexible functional dependence of the outcome variable on the covariates^8^.

Growth mixture model (GMM), latent class growth analysis (LCGA), and longitudinal latent class analysis (LLCA) are some approaches for GLMMs. These approaches parametrically model the trajectories of individuals and assume that there exists a latent variable consisting of diverse classes that causes heterogeneity through time^9–13^.

Generalized additive mixed models (GAMMs) are a solution to these issues. They consider nonparametric functions of covariates and random effects for correlation in longitudinal data using smoothing splines^7^. Combining GAMMs with latent class analysis introduces a nonparametric approach called the smoothing mixture model (SMM), which allows smoothing functions of trajectories and provides class membership^14^.

Maternal weight gain is one of the risk factors associated with adverse pregnancy outcomes for both mother and child^15–17^. There are some estimated thresholds for the trend of weight gain in pregnant women for healthier pregnancy period. However, heterogeneous populations in terms of different biological, social and economic factors make these specific measures controversial. Classifying individuals into subgroups through trajectories of longitudinal data provides confirmed phenotypes^18–21^.

Insufficient weight gain has been linked with increased risks of preterm birth, SGA and LBW, while excessive weight gain has been associated with macrosomia, GDM, preeclampsia, preterm birth and infant mortality^22^.

The aim of this study is to identify latent longitudinal trajectories of maternal weight during the pregnancy period and estimate their associations with adverse pregnancy outcomes. This is the first study estimating maternal weight trajectories during pregnancy with nine recorded repeated measures using the SMM approach. We applied the new nonparametric statistical approach SMM which has more flexibility than other, parametric methods and also provides more accurate group membership classification using modified expectation–maximization algorithm. SMM approach is straightforward to implement using the “gamm4” R-package, and it can also be applied to time-varying covariates and longitudinal data with any exponential family distribution, such as normal, Bernoulli, and Poisson.

## Materials and methods

This is a secondary study carried out on the data from the Khuzestan Vitamin D Deficiency Screening Program in Pregnancy^23^. Out of 1800 pregnant women referred to the health centers of Masjed-Soleyman and Shooshtar (Khuzestan Province, Iran), 877 residents of Shooshtar city were selected for this study, whose weights during the 9 months of pregnancy were available. The eligibility criteria involved age range 18–40 years, gestational age < 14 weeks, singleton pregnancy, not consuming multivitamins containing 400 international units per day (IU/d) of D3, and no previous history of chronic diseases like diabetes, hypertension, renal dysfunction, or liver disease. Participants signed a written informed consent during recruitment covering all trial procedures and data collection. It was emphasized that participation in the study was voluntary and they were free to withdraw from the study at any time. Preterm birth, preeclampsia, GDM, icterus, abnormality, and NICU admission were considered as the outcome measures. Composite maternal events (having at list one of the outcomes of preterm birth, preeclampsia or GDM) and composite neonatal events (icterus, abnormality, or NICU admission) were calculated as well. Maternal age, gestational age, gravidity as well as estimated trajectories of maternal weight were considered as the potential covariates.

Pregnancy care checklist was filled out with repeated referrals to pregnancy care clinic for all pregnant women according to the instructions of the Ministry of Health. Referrals for pregnant women were done according to the mentioned instructions in 6–10 weeks (or 11–15 weeks), 20 weeks (or 21–25 weeks), 26–30 weeks, 31–34 weeks, 35–37 weeks, 38 weeks, 39 weeks, 40 weeks or 41 weeks. In order to determine the reliability of the scale, the weight of 15 people was measured twice, and the intra-class correlation coefficient (ICC) was estimated as 0.95. Calibrated Seca model 755 vertical dial scales made in Germany were applied for all measurements in each visit.

### Ethical considerations

Written informed consent was obtained from all participants, and the study was approved by the Ethics Committee of the Research Institute of Endocrine Sciences (approval no. 10ECRIES25/10/92). This study is registered in the Iranian Registry of Clinical Trials (code no. IRCT2014102519660N1). We also confirm that all methods were performed in accordance with the relevant guidelines and regulations^24^.

## Method

A modified expectation–maximization (E–M) algorithm was applied to classify participants into homogeneous groups. Group assignment was accomplished by iterating the M and E steps. In the first iteration, participants were assigned to a predefined number of groups according to the mean value of trajectories. In the M step, the GAMMs with a smoothing function of time were fitted for all groups and achieved predicted trajectories from all GAMMs for each individual. In the E step, participants were reassigned to the group for which the estimated trajectory was the most similar to the observed one. The modified E-M algorithm was iterated until group memberships were no longer changed and the sum of the largest log-likelihood (LL) for all individuals remained the same. The Bayesian information criterion (BIC) was applied to compare model fit assuming different numbers of groups and to find the optimum number of classes. SMM defined as the nonparametric GAMM with smoothing spline for the time effect was fitted using the “gamm4” package (version 0.2–6) in R 4.0.2.^14^. Then the logistic regression model was fitted to estimate crude and adjusted odds ratio (OR) of adverse pregnancy outcomes for each pregnancy weight phenotype defined by the optimum number of longitudinal trajectories using “glm” function in R 4.0.2. The rule of thumb of a p-value lower than 0.2 was applied in the univariate logistic regression to detect potential confounding variables.

## Results

Table 1 provides descriptive characteristics of the 877 included individuals. Normality distribution of data was checked using Kolmogorov–Smirnov and Shapiro–Wilk which showed non-normal distribution of weights (p-value < 0.001). Therefore, smoothing functions in SMM use nonparametric penalized splines, resulting in highly flexible trajectories.

**Table 1:** Descriptive statistics of variables.

Continuous variables	Mean (SD)	Median (Q1–Q3)
Age	29 (5)	29 (25–32)
Gestational age	38 (1)	38 (37–39)
Gravidity	2 (1)	2 (1–3)
Maternal weight 1st month	64.6 (7.1)	64 (59–70)
Maternal weight 2nd month	66.2 (7.1)	65.5 (60–71.5)
Maternal weight 3rd month	67.7 (7.1)	67 (62–73)
Maternal weight 4th month	69.3 (7.1)	68.5 (64–74.5)
Maternal weight 5th month	70.9 (7.1)	70 (65.5–76)
Maternal weight 6th month	72.5 (7.2)	72 (67–78)
Maternal weight 7th month	73.3 (7.3)	73 (68–78.5)
Maternal weight 8th month	73.4 (7.7)	73 (68–79)
Maternal weight 9th month	73.5 (7.4)	73 (68–79)

Discrete variables	N (%)	
Icterus	251(28.8)	
Abnormality	29(3.3)	
NICU admission	148(16.9)	
Preterm delivery	120(13.7)	
Preeclampsia	138(15.7)	
Gestational diabetes	53(6.1)	
Composite maternal events^#^	240(27.4)	
Composite neonatal events^##^	288(32.9)	

^#^Composite maternal events: Preterm, Preeclampsia, GDM.

^##^Composite neonatal events: Icterus, Abnormality, NICU admission.

**Figure 1:**
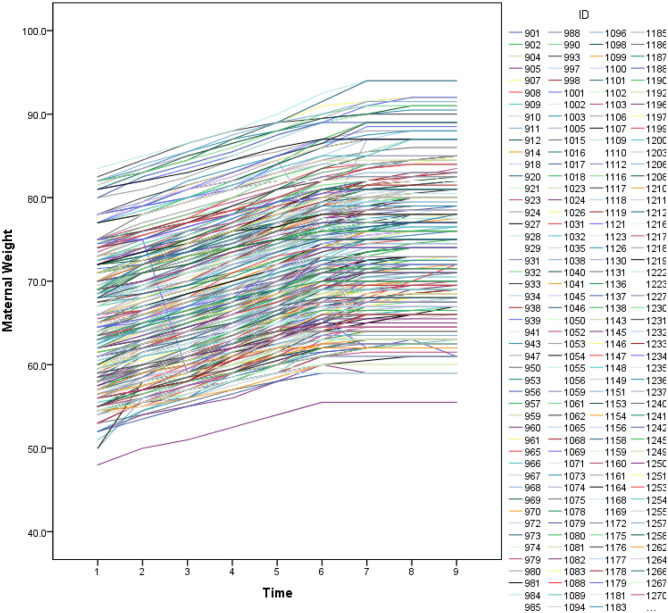
Spaghetti plot of 50% randomly selected participants’ maternal weight trajectories during pregnancy.

Figure 1 illustrates the spaghetti plot of maternal weight trajectories for fifty percent of participants during nine months of pregnancy. Nearly most of the participants in this study gained weight with similar growth lines, which suggests lower individual-specific random effects and thus high to medium separation with relatively low heterogeneity in trajectories.

Table 2 shows the summary results of SMM estimating different classes of maternal weight longitudinal trajectories. Estimated BIC and LL for two, three, four and five classes of trajectories were (BIC: 32,485.4, LL: − 16,193.8), (30,528.3, − 15,189.1), (30,211.9, − 15,014.3), (29,626.5, − 14,684.3), respectively.

**Table 2:** Summary results of smoothing mixture model (SMM) to estimate different classes of maternal weight longitudinal trajectories.

Number of trajectory classifications	BIC	LL	Trajectory median (IQR)
Two classifications	32,485.4	− 16,193.8	65.0 (6)
			77.0 (7)
Three classifications	30,528.3	− 15,189.1	63.0 (6)
			71.5 (6)
			79.5 (6)
Four classifications	30,211.9	− 15,014.3	62.0 (6)
			68.5 (6)
			76.0 (6)
			84.5 (6)
Five classifications	29,626.5	− 14,684.3	60.0 (6.5)
			65.0 (6)
			70.0 (5.5)
			76.5 (6)
			84.5 (6.5)

**Figure 2:**
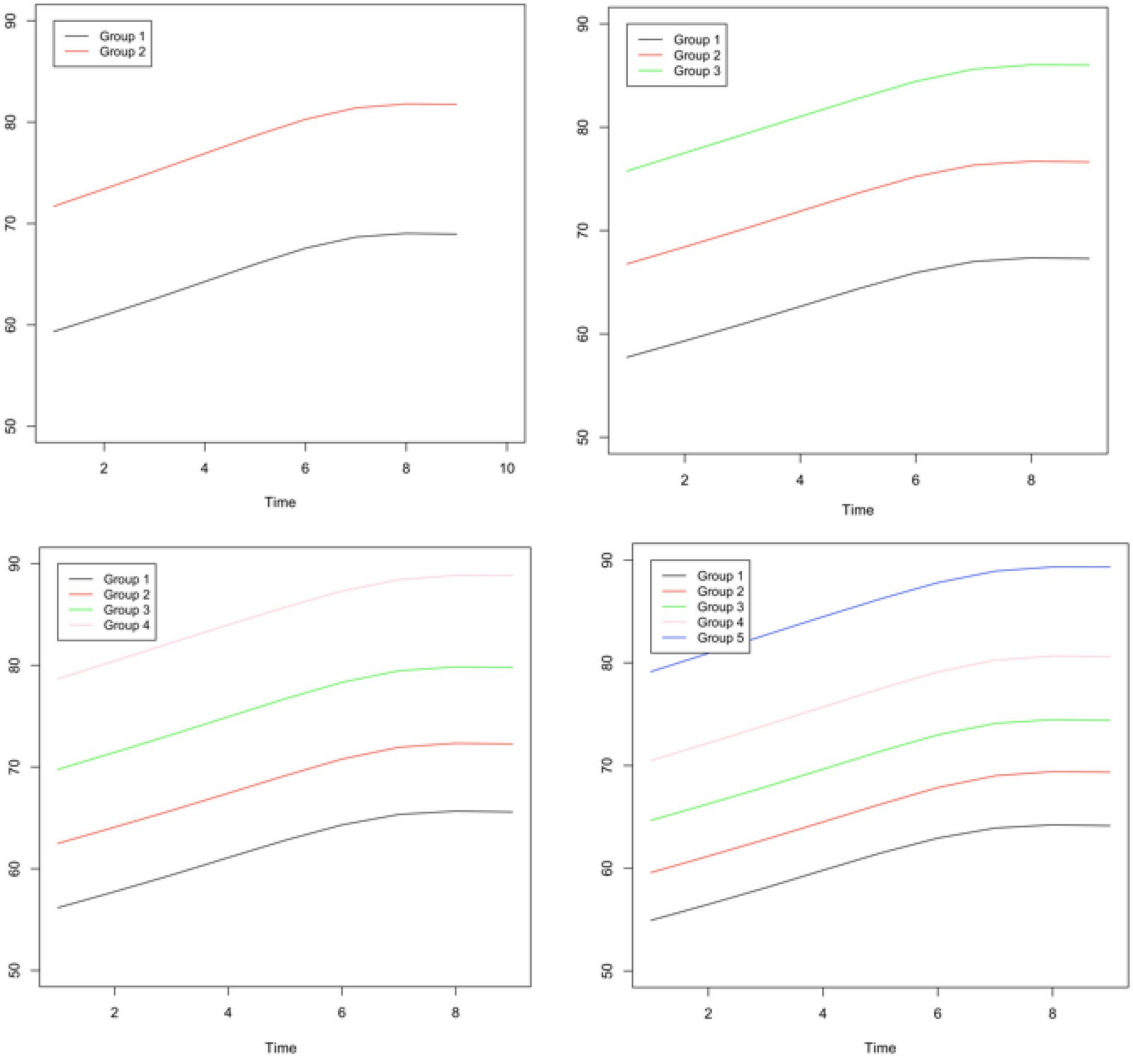
Predicted trajectories for maternal weight during 9 months of pregnancy using smoothing mixture model (SMM).

The minimum value of BIC and LL is considered as the acceptance criteria for the best classification. There are no significant differences between three and five classifications, so we chose three groups as a parsimonious model that captures a large amount of variation in trajectories (Fig. 2). Maternal weight trajectories were named as Low (n = 337 [38%], median weight = 63 kg, IQR = 6 kg), Medium (n = 319 [36%], 71.5 kg, 6 kg) and High (n = 221 [26%], 79.5 kg, 6 kg).

The cross tabulation of initial grouping (based on baseline maternal weight) and five-group classification (based on SMM predicted model of class membership) is illustrated in Table 3. It shows the number of concordances and discordances equal 29% and 71%, respectively.

**Table 3:** Cross table of initial grouping based on baseline maternal weight and five-group classification based on SMM predicted model trajectories to illustrated number of concordances and discordances.

		Five-group classification based on SMM Predicted model trajectories					
		1.00	2.00	3.00	4.00	5.00	Total
Initial grouping based on baseline MW	1	127	174	2	0	0	303
	2	0	43	173	2	0	218
	3	0	1	19	228	0	248
	4	0	0	1	37	38	76
	5	0	3	0	1	28	32
	Total	127	221	195	268	66	877

Crude OR for icterus, preterm delivery, NICU admission and composite neonatal events shows higher risks in trajectory 1 (low weight) compared to trajectory 2 (medium weight) by 69% (OR = 1.69, 95%CI 1.20, 2.39), 82% (OR = 1.82, 95%CI 1.14, 2.87), 77% (OR = 1.77, 95%CI 1.17, 2.43), and 85% (OR = 1.85, 95%CI 1.38, 2.76), respectively. The results were consistent after adjusting for maternal age, gestational age and gravidity for icterus, NICU admission and composite neonatal events. No significant results were found for trajectory 3 (high weight) versus trajectory 2 (Table 4).

**Table 4:** Results of logistic regression model to examine associations of the three identified trajectories of maternal weight with risk of adverse pregnancy events.

Outcomes	Crude OR (95%CI)	^&^Adjusted OR (95%CI)
Icterus	1.44 (0.97, 2.23)	^@^ 1.49 (0.95, 2.11)
	1.69 (1.20, 2.39) *	^@@^ 1.63 (1.06, 2.51) *
Preterm delivery	1.11 (0.65, 1.92)	2.10 (0.57, 7.75)
	1.82 (1.14, 2.87) *	1.09 (0.34, 2.97)
Preeclampsia	1.19 (0.75, 1.89)	0.86 (0.47, 1.50)
	0.93 (0.70, 1.64)	0.92 (0.59, 1.70)
Abnormality	2.56 (0.66, 6.45)	2.06 (0.65, 1.72)
	1.50 (0.25, 3.74)	1.47 (0.67, 4.12)
NICU admission	1.09 (0.64, 1.78)	1.03 (0.54, 1.96)
	1.77 (1.17, 2.43) *	1.73 (1.03, 2.99) *
GDM	1.21 (0.60, 2.44)	1.34 (0.53, 3.44)
	0.96 (0.55, 1.84)	1.13 (0.55, 2.30)
Composite maternal events	1.18 (0.86, 1.75)	1.28 (0.93, 1.72)
	1.25 (0.86, 1.73)	1.15 (0.76, 1.89)
Composite neonatal events	1.48 (0.99, 2.12)	1.44 (0.99, 2.11)
	1.85 (1.38, 2.76) *	1.85 (1.32, 2.58) *

^@^Trajectory 3 versus Trajectory 2.

^@@^Trajectory 1 versus Trajectory 2.

^&^Logistic regression model adjusted by maternal age, gestational age and gravidity.

*Significant level < 0.05.

## Discussion

Various classes of methods have been introduced for the identification of latent trajectories in health data sciences. In this study, we identified three latent trajectories for maternal weight during pregnancy and then named them as low, medium and high weight trajectories using SMM. To the best of our knowledge, this is the first study estimating latent maternal weight trajectories during the nine months of pregnancy and assessing their association with adverse maternal and neonatal events. Results of this study showed that low compared to medium weight trajectories significantly increased the risk of icterus, preterm delivery, NICU admission and composite neonatal events by 69%, 82%, 77% and 85%, respectively.

Our study results were consistent with many studies showing that low maternal weight gain during pregnancy is considered a significant risk factor associated with adverse pregnancy events such as preterm birth, low birth weight, maternal delivery complications, and prematurity^25–28^.

Different classification schemes for maternal weight gain have been recommended in the literature applying various statistical methods such as detecting cutoff points, threshold estimation, and clustering approaches which have specific algorithms to classify individuals^21,29–31^. However, subgrouping individuals using longitudinal information and statistical methods which introduce tools to evaluate individual variation, and identifying subgroups within the population known as latent classes following distinct developmental paths over time named trajectories can be more accurate^32–34^. There are some examples in the literature which clarify this issue better. A study on 4436 pairs of mothers and their children in the National Longitudinal Survey of Youth, identified longitudinal trajectories of maternal weight from before pregnancy through the postpartum period using latent-class growth modeling and then assessed the relationship between the trajectories and offspring obesity. They also compared results of the maternal weight trajectories based on either latent-class results or recommendations to classify trajectory groups which both approaches showed a low risk of child obesity in the lowest maternal weight trajectory group^35^.

Latent class growth analysis and the growth mixture model are two popular statistical approaches widely applied for identifying participants within a population with heterogeneous longitudinal trajectories. However, both methods parametrically model the trajectories of individuals leading to limited flexibility in modeling trajectories^36–38^. Alternatively, a mixture model allowing for smoothing functions of longitudinal trajectories overcomes this issue by providing a flexible means to model longitudinal count or non-normal data and also considering the time-varying covariates^14^. Generally, SMM uses nonparametric functions of covariates and applies random effects to account for correlation in longitudinal data and combines it with latent class analysis.

Results from our study suggest that individuals with high, medium and low weight at baseline share similar growth patterns, and individuals with high weight in the conception of pregnancy are highly likely to remain obese at delivery. One justification for these results could be the nature of the data. In this study, nearly most of the participants gain weight with a similar growth pattern. We illustrated it with the spaghetti plot of 50% of randomly selected participants. However, there are some discordances between the initial group membership obtained by the baseline maternal weight and the model group membership indicated by the estimated trajectories. Therefore, SMM well differentiated individuals based on their pattern of weight gain over time. Our results are in line with some other studies which studied maternal weight trajectories^39,40^.

Our study also showed that beginning pregnancy in an overweight state leads to gaining more excessive weight during pregnancy. There is consistent evidence that starting pregnancy at a high weight and continuing it with gaining excessive weight during pregnancy increases the risk of pregnancy events^41,42^.

For more elaboration, the relationship between hypertensive disorders such as preeclampsia and the weight of women entering pregnancy is well established; however, the association between these conditions and increased maternal weight gain is less clear^43,44^. A rationale for this is the mutual relationship between preeclampsia and weight gain, in a way that preeclampsia decreases the expansion in maternal intravascular volume which may also affect weight gain in early gestation. In addition, preeclampsia causes increased vascular permeability and decreased plasma oncotic pressure, which may lead to increased edema and excessive weight gain in late gestation^45^.

Several studies also investigated the impact of maternal obesity on NICU admission^46^. Although the reason for the neonatal complications in obese women is unknown, some pathological issues could be related to increased maternal pelvic soft tissue, difficulty in estimating the fetal weight, and intrapartum complications like the inability to adequately monitor the fetus and contractions^47–49^.

On the contrary, insufficient weight gain during pregnancy is a controversial issue which is associated with higher risks of some obstetrical complications such as prematurity and small-for-gestational age^22^. Studies have shown that nearly 23% of pregnant women are out of the recommendations with insufficient gestational weight gain. Our results are in agreement with published studies showing an increased risk of preterm birth in case of insufficient weight gain^50^.

In summary, the results of our study revealed a U-shaped form of associations which means that extreme levels of maternal weight gains, namely low and high trajectories, are important risk factors for most pregnancy complications^51,52^. Therefore, to minimize the risk of adverse pregnancy events, the optimum place for pregnant women could be in the middle of this growth curve.

The main strength of our study was the use of SMM as the accurate modelling approach for non-normal longitudinal data to estimate latent maternal weight trajectories which indicate the pattern of weight gain. Smoothing functions in GAMM part of this approach use nonparametric penalized splines; therefore, it can identify highly flexible trajectories without the issue of overfitting^14^.

In addition, we applied this approach in a longitudinal data with a 9-month repeated measure for maternal weight in order to capture the accurate patterns of change, while a limit on analyses which weakens the evidence was the calculation of maternal weight gain in observational studies, mainly in retrospective cohorts. Many studies considered total gestational weight gain, which complicates a comparison between pregnancy adverse outcomes.

Furthermore, we considered both maternal and neonatal outcomes and assessed the association between trajectories and events.

Considering BMI as another measure of weight index could be of more interest. However, not recording BMI in our dataset was a limitation of our study, which can be ignored due to the nature of longitudinal data and the within-subject variations. Baseline maternal weight was measured based on the inclusion criteria of women with gestational age lower than 14 weeks, and randomized control trial design of the study could control the effect of confounding variables. In addition, despite the potential advantages for highly flexible modelling of trajectories and convenient application to data with non-normal distributions, one of the limitations of SMM is using BIC as a criterion. Ding et al. (2020) discussed that SMM is inclined to detect too many groups in case of low separation among groups, in which trajectories with low separation had larger individual-specific random effects; however, it performed well when there was medium to high separation with relatively low heterogeneity in trajectories. Nevertheless, this is also a common issue for all types of mixture models^14^. Moreover, although this is a very feasible model in terms of statistical technique by using nonparametric smoothing spline for flexible modelling of trajectories of non-normally distributed outcomes, SMM applied a modified algorithm in the M step by assigning an individual to only one group with the highest membership probability and ignored uncertainty in class membership which is another limitation of this model.

## Conclusion

Latent class trajectories of maternal weight can be accurately estimated using SMM. SMM is a powerful means for researchers to appropriately assign individuals to their class. The U-shaped curve of association between maternal weight gain and risk of maternal complications reveals that the optimum place for pregnant women could be in the middle of the growth curve to minimize the risks. Low maternal weight trajectory compared to high had even a significantly higher hazard for some neonatal adverse events. Therefore, appropriate weight gain is critical for pregnant women.

## Data Availability

The original contributions presented in the study are included in the article. Further inquiries can be directed to the corresponding author.
